# Computer-Mediated Communication to Facilitate Synchronous Online Focus Group Discussions: Feasibility Study for Qualitative HIV Research Among Transgender Women Across the United States

**DOI:** 10.2196/12569

**Published:** 2019-03-29

**Authors:** Andrea L Wirtz, Erin E Cooney, Aeysha Chaudhry, Sari L Reisner, American Cohort To Study HIV Acquisition Among Transgender Women

**Affiliations:** 1 Department of Epidemiology Center for Public Health and Human Rights Johns Hopkins Bloomberg School of Public Health Baltimore, MD United States; 2 Division of General Pediatrics Boston Children’s Hospital Boston, MA United States; 3 Pediatrics Harvard Medical School Boston, MA United States; 4 Department of Epidemiology Harvard TH Chan School of Public Health Boston, MA United States; 5 Fenway Health The Fenway Institute Boston, MA United States

**Keywords:** transgender, qualitative research, formative research, technology

## Abstract

**Background:**

Novel, technology-based methods are rapidly increasing in popularity across multiple facets of quantitative research. Qualitative research, however, has been slower to integrate technology into research methodology. One method, computer-mediated communication (CMC), has been utilized to a limited extent for focus group discussions.

**Objective:**

This study aimed to assess feasibility of an online video conferencing system to further adapt CMC to facilitate synchronous focus group discussions among transgender women living in six cities in eastern and southern United States.

**Methods:**

Between August 2017 and January 2018, focus group discussions with adult transgender women were conducted in English and Spanish by research teams based in Boston, MA, and Baltimore, MD. Participants were sampled from six cities: Baltimore, MD; Boston, MA; New York, NY; Washington, DC; Atlanta, GA; and Miami, FL. This was formative research to inform a technology-enhanced cohort study to assess HIV acquisition among transgender women. This analysis focused on the methodologic use of CMC focus groups conducted synchronously using online software that enabled video or phone discussion. Findings were based on qualitative observations of attendance and study team debriefing on topics of individual, social, technical, and logistical challenges encountered.

**Results:**

A total of 41 transgender women from all six cities participated in seven online focus group discussions—five English and two Spanish. There was equal racial distribution of black/African American (14/41, 34%) and white (14/41, 34%) attendees, with 29% (12/41) identifying as Hispanic/Latina ethnicity. Overall, 29 of 70 (41%) eligible and scheduled transgender women failed to attend the focus group discussions. The most common reason for nonattendance was forgetting or having a scheduling conflict (16/29, 55%). A total of 14% (4/29) reported technical challenges associated with accessing the CMC focus group discussion. CMC focus group discussions were found to facilitate geographic diversity; allow participants to control anonymity and privacy (eg, use of pseudonyms and option to use video); ease scheduling by eliminating challenges related to travel to a data collection site; and offer flexibility to join via a variety of devices. Challenges encountered were related to overlapping conversations; variable audio quality in cases where Internet or cellular connection was poor; and distribution of incentives (eg, cash versus gift cards). As with all focus group discussions, establishment of ground rules and employing both a skilled facilitator and a notetaker who could troubleshoot technology issues were critical to the success of CMC focus group discussions.

**Conclusions:**

Synchronous CMC focus group discussions provide a secure opportunity to convene participants across geographic space with minimal time burden and without losing the standardized approach that is expected of focus group discussions. This method may provide an optimal alternative to engaging hard-to-reach participants in focus group discussions. Participants with limited technological literacy or inconsistent access to a phone and/or cellular data or service, as well as circumstances necessitating immediate cash incentives may, however, require additional support and accommodation when participating in CMC focus group discussions.

## Introduction

Novel, technology-based methods are rapidly increasing in popularity across multiple facets of quantitative HIV research. For several years now, technology has been integrated into sampling and recruitment methods via the use of online advertisements on social media and dating apps, electronic and short message service (SMS) text messaging surveys for data collection, online incentive systems, and electronic gift cards. Outside of HIV research, several large cohorts, such as the National Institutes of Health’s (NIH) Precision Medicine Initiative Cohort [1] and the Black Women’s Health Study [2], recruit and follow over 50,000 to 1 million participants across the United States using predominantly technology-based methods. HIV research is keeping with these trends, enrolling large samples of participants for behavioral surveys [3-5] and intervention research [6,7]. The development of SMS text messaging and mobile phone apps has become an increasingly common method to provide and support prevention and care interventions [8,9], while gaming methods have been incorporated to keep participants, particularly youth, engaged in research activities and to promote and maintain health behaviors [10,11]. The combined benefits of improving overall study efficiency, potentially decreasing study costs associated with reducing space and staff needs for data collection, and improving convenience for participants suggest that integration of technology into research practices will continue to emerge and evolve.

Qualitative research, however, has been arguably slower to integrate technology into its research methodology. One method, computer-mediated communication (CMC), has been utilized to a limited extent for focus group discussions. CMC, or *online* focus groups, has been used with populations that include, but are not limited to, cancer survivors [12,13], transplant patients [14], adolescents [15], gay and bisexual men [16], and transgender men and women [17,18]. This method provides a platform in which participants may join a virtual group to respond to a series of open-ended questions that follow a typical semistructured guide.

Previously, CMC focus group discussions have used online discussion forums in an asynchronous format that allows participants to type responses to posted questions [16]. In the asynchronous format, a group of participants may respond to the questions and to prior posts by other participants, but not all participants may be participating at the same time. The limited research of this method has suggested that CMC provides multiple benefits in terms of reducing cost and barriers associated with finding an ideal time and physical space that is convenient for participants to meet. Comparisons of in-person versus CMC focus groups have found that visual anonymity and perceived distance across the Internet stimulates group discussion and disclosure, allowing for greater sharing of ideas during CMC [19]. While typed responses provide more direct responses to questions (ie, less digression from topics) and candid sharing of sensitive information in an anonymous and confidential environment [16], discomfort with typing and low literacy levels may prohibit some participants from fully describing their viewpoints or experiences. Conversely, synchronous CMC focus groups that use audio or video options allow participants to join the discussion at the same time and respond to the question, as well as to each other’s comments, in real time. Online meeting platforms, which allow participants to join securely and free of charge from a variety of online or telephone mediums, may solve some of the initial limitations of typed, asynchronous CMC qualitative research, while still providing the same benefits.

This study aimed to assess feasibility of an online conferencing system to adapt CMC methods to facilitate focus group discussions in a synchronous audio and video format among transgender women living in six cities in eastern and southern United States.

## Methods

### Setting

Between August 2017 and January 2018, a total of seven synchronous focus group discussions with adult transgender women were conducted by research teams in Boston, MA, and Baltimore, MD. Participants were sampled from across six cities, including Baltimore, MD; Boston, MA; New York, NY; Washington, DC; Atlanta, GA; and Miami, FL. The focus group discussions were conducted as formative research to develop and implement a technology-enhanced cohort study to assess HIV acquisition among transgender women in these cities.

Synchronous CMC focus groups were selected for use with this study population, given past observations of challenges faced by participants in terms of transportation to and from the physical location of the focus group discussions [20]. Interest in convening focus groups across the six study cities further supported the decision to use synchronous CMC methods.

### Recruitment, Eligibility Screening, and Enrollment

To reach a diverse sample, participant recruitment was conducted via multiple avenues, including word-of-mouth, advertisements shared at or by local community-based organizations and clinics, and advertisements shared via social media. Candidate participants were informed that the study was to “understand more about HIV and other health concerns among transgender women across the US.” Participants were informed that the purpose of this study was to obtain their feedback, opinion, and suggestions on the methods that would be used in an upcoming study among transgender women.

Participants who were interested in joining a focus group contacted the study coordinator who screened participants by phone to assess eligibility. Participants were deemed eligible if they met the following criteria: aged 18 years or older; reported an identity along the trans feminine spectrum, which included reporting being both assigned male sex at birth and reporting transgender, gender nonconforming, or female gender identity; and no prior participation in the focus group discussion. We used maximum variation sampling [21] in an effort to ensure representation across geographic site, race and ethnicity, and age.

Focus group discussions were available in English or Spanish languages, and the screening and consent processes followed the individual’s preferred language. Eligible candidates consented and were then scheduled for a focus group at a time and day that was convenient to them. The verbal consent script informed participants of the purpose of the study, the reason they were invited to participate, and the risks and benefits to participants. Participants were informed that they could use their real name or a nickname or alias during the focus group discussion to protect their privacy and could opt to use a Web camera during the focus group but were informed that this could reduce their privacy.

Study staff informally assessed technological literacy during initial screening telephone calls. Study staff were therefore able to tailor instructions and troubleshooting tips based on participant comfort and preference, with the intention of including participants with varying levels of technological literacy. Participants were provided with verbal instructions via the telephone and were emailed instructions on how to join via multiple platforms. Simple instructions that were augmented by screenshots and other images described how the participant could join the focus group by cell phone, landline, mobile app, or via Web-based connection. Email and text message reminders were also sent to participants the day of the CMC focus group to remind them of their upcoming discussion. Additional effort focused on providing support to participants who appeared to have lower levels of technological literacy or lower levels of comfort with technology. For these participants, staff members often recommended calling into the focus group discussion, rather than use a computer or tablet.

### Focus Group Implementation

Groups were comprised of 5-10 participants across geographic locations. Focus group discussions lasted approximately 60-90 minutes and were led by a facilitator with the support of a notetaker from the study team. The notetaker played the traditional *notetaker* role utilized in focus group discussions, but also provided technical support during the focus group discussion. This included making sure that participants were able to securely connect to the conference system by telephone or computer, including ensuring that speakers and microphones were enabled; troubleshooting any login challenges; reminding participants that they had the option to use their name or alias, as well as to enable or disable their video depending on their privacy preferences; and documenting names of participants to ensure that the appropriate study incentive was sent following the discussion. Focus groups were implemented using the Zoom online meeting platform (Zoom Video Communications, Inc), which is a Health Insurance Portability and Accountability Act (HIPAA)-compliant system that provides end-to-end encryption and optional audio and video recording [22]. Once connected to the group, participants could communicate individually with study staff using the private message option within the system for any additional support.

The focus group facilitator specified ground rules at the start of the discussion; reminded participants of key aspects of the consent form, including consent to record; and verified group consent to record before the audio recording began. The focus group discussion was then implemented using a semistructured guide to ask open-ended questions that targeted the primary research questions of interest. Following the conclusion of the focus group discussions, participants were sent an Amazon gift card in the amount of US $40. After encountering challenges to enrolling participants in Miami, FL, we subsequently revised our methodology to offer cash incentives.

Focus group guides were developed to gain insight from transgender women community members for the development of the subsequent cohort. The guide was developed to collect information on the feasibility, acceptability, and potential barriers of various recruitment and enrollment methods, as well as study branding and marketing.

### Analysis

All focus group discussions were recorded via the Zoom software platform; audio recordings were submitted to a professional transcription company to be transcribed. Transcripts were reviewed for accuracy by staff members who were present during the focus group. Focus group transcripts were complemented by detailed notes and debriefing meetings with study staff following the discussions. Trained qualitative analysts dually coded the transcripts using NVivo qualitative analysis software (QSR International) and met to discuss agreement and resolve discrepancies in coding. Spanish transcripts were coded in Spanish by Spanish-speaking staff and key quotes were translated into English.

This analysis focuses on the methodologic use of CMC focus groups conducted synchronously using online software that enables video or phone discussion for the purpose of informing future use of this method. Findings are based on qualitative observations of attendance and study team debriefing on topics of individual, social, technical, and logistical challenges encountered by the study team or by participants.

### Ethical Review

This study was approved by the Institutional Review Board at the Johns Hopkins School of Public Health. The collaborating partners, The Fenway Institute and Boston Children’s Hospital, ceded review to the Johns Hopkins School of Public Health as the prime recipient of the funding. The study was also supported by a community review provided by a Community Advisory Board comprised of transgender women community members from across the six participating cities.

## Results

### Sample

A total of 41 transgender women, of the 70 who were eligible, participated in seven online focus group discussions—five English and two Spanish—between August 2017 and January 2018. There was equal racial distribution of black/African American (14/41, 34%) and white (14/41, 34%) attendees, with 29% (12/41) identifying as Hispanic/Latina ethnicity. Participants were from all six cities, though slightly more from Baltimore and Washington, DC, participated in the discussions (9/41, 22% and 11/41, 27%, respectively) (see Table 1).

A total of 29 of 70 (41%) eligible and scheduled transgender women failed to attend the focus group discussions. Over half (16/29, 55%) were black/African American and 34% (10/29) were mixed race or other. Nonattendees were fairly distributed across the cities, with the exception that a higher proportion were from Washington, DC (see Table 1). The most common reason for nonattendance was forgetting or having a scheduling conflict (16/29, 55%). A total of 14% (4/29) reported technical challenges associated with accessing the CMC focus group discussion.

### Benefits

We observed several benefits associated with the CMC focus group discussions. The use of an online forum facilitated geographic diversity by bringing together participants from multiple cities, which increased the racial and ethnic diversity of the sample. The geographic distance between participants, coupled with the ability to use pseudonyms, and the option to use video provided additional measures for anonymity and study privacy. We observed that a few participants, however, had prior social connections despite geographic distances, particularly through online venues and social networks. The use of CMC also eased scheduling by eliminating challenges related to participants’ travel to a data collection site. These benefits were augmented by the flexibility of the system to allow participants to join the discussion via telephone (ie, cell or landline), mobile phone or tablet (ie, through an app), or computer.

**Table 1:** Focus group participant characteristics, stratified by attendance.

Characteristic		Attendees (N=41)	Nonattendees (N=29)	Total eligible participants (N=70)
Age in years, mean (SD)		39.3 (13.6)	40.1 (12.9)	39.6 (13.2)
**Race/ethnicity, n (%)**				
	White	14 (34)	3 (10)	17 (24)
	Black	14 (34)	16 (55)	30 (43)
	Asian	2 (5)	0 (0)	2 (3)
	Hispanic/Latina	12 (29)	10 (34)	22 (31)
	More than one race or other	11 (27)	10 (34)	21 (30)
**City, n (%)**				
	Atlanta	7 (17)	4 (14)	11 (16)
	Baltimore	9 (22)	4 (14)	13 (19)
	Boston	4 (10)	4 (14)	8 (11)
	New York City	5 (12)	3 (10)	8 (11)
	Miami	5 (12)	4 (14)	9 (13)
	Washington, DC	11 (27)	10 (34)	21 (30)
**Reasons for not attending, n (%)**				
	Forgot or scheduling conflict	N/A^a^	16 (55)	N/A
	Technological challenge	N/A	4 (14)	N/A
	Unknown	N/A	9 (31)	N/A

^a^N/A: not applicable.

### Challenges

Several challenges were encountered during CMC focus group discussions, but they were predominantly minor in nature. These tended to include overlapping conversations and variable audio quality. Because some participants elected to keep their video turned off, it was easy for multiple participants to speak at the same time. Most participants immediately recognized when this occurred and would often take turns allowing each other to speak. CMC focus group discussions were ultimately facilitated in approximately the same way that in-person focus group discussions are facilitated and the facilitators made a point to refer back to anyone who had not been able to provide a response. Audio quality varied in cases where Internet or cellular connection was poor or when participants situated their computer or phone in a way such that the microphone was muffled. These were rare events and tended to be resolved when it was brought to the participant’s attention that they could not be heard. Ground rules were established at the beginning of the discussions to remind participants to allow each other to speak and to keep their phones or computers muted when not speaking.

### Additional Considerations

Our experience with Spanish-speaking participants in Miami provided a unique challenge. Upon screening and enrollment, most candidate participants informed study staff that they did not have a phone or computer by which they could join the CMC focus groups. Ultimately, it was decided that our collaborating partners in Miami would host the focus group discussion in their office where trained research staff from Boston and Baltimore were able to connect via the video conference software. This situation proved to be the most difficult for the use of CMC focus group discussions. In prior CMC focus groups, it had been possible to view the facial expressions and body language of participants who elected to use video, and individual use of phones and computers permitted relatively clear and audible discussions. However, the setup of the camera limited the view of some participants who were sitting in the group. The setup also made it more difficult to hear participants who were sitting further away from the computer. Ultimately, the CMC focus group was able to be implemented and provided useful formative information for this population. After discussion with local staff in Miami, it was understood that many of the participants did, indeed, have cell phones, which they brought to the discussion, but simply preferred to receive cash rather than an Amazon gift card incentive.

## Discussion

### Principal Findings

The use of CMC focus group discussions provides an efficient and secure opportunity to convene participants across geographic space. In this case, the use of CMC focus group discussions facilitated geographic diversity, empowered participants to control their privacy during the focus group discussion, and provided flexibility in scheduling by eliminating challenges related to traveling to a data collection site. These benefits are particularly important for transgender women and other hard-to-reach populations who have multiple competing priorities and who may prefer more privacy in research participation. The benefits and challenges of CMC focus group discussions were balanced against the benefits and challenges that are associated with in-person focus group discussions (see Table 2). With consideration to unique aspects of CMC focus group discussions, we found that CMC focus group discussions are feasible to implement in overcoming challenges and barriers typically associated with in-person focus group discussions and with no apparent bearing on data or recording quality. The personal skills of facilitators and notetakers are as crucial to successful implementation of CMC focus group discussions as they are in person [23]. While the Zoom conference platform was used for these focus group discussions, any electronic platform that meets the following criteria may suffice for this purpose: allows synchronous audio and/or video communication, provides appropriate security measures, and is free of charge to participants.

In the context of this particular study, the greatest benefits of CMC focus group discussions were the flexibility in scheduling and the geographic reach offered by this method. Rarely are there situations in which busy participants can find a mutually agreeable time to participate in a focus group. CMC focus group discussions, however, reduced the burden of identifying a mutually agreeable location for participants to meet, as well as reduced time for transport to and from the location of the discussion. For transgender women, this may have the added effect of reducing travel time and costs, given expense of parking and that use of public transportation can add an additional 2-hour round trip to attend an hour-long focus group discussion in these cities. CMC focus groups also obviate security risks that are associated with traveling to unfamiliar locations and potential exposure to stigmatization and violence among this population [24]. Participants further seemed to enjoy speaking to others from different cities and these cross-city discussions provided the opportunity to assess and discuss differences across geographic locations.

**Table 2:** Comparison of traditional, in-person focus group discussions with CMC^a^ focus group discussions and considerations for implementation of CMC focus groups.

Study component	Traditional, in-person focus group discussions	CMC focus group discussions	Considerations for CMC focus group discussions
Geographic distribution	Constrained to local setting	Can be conducted across geographic distance, pulling participants and staff across distances	Time differences
Scheduling	Requires mutually agreeable date, time, and location; additional time needed to travel to and from study site	Requires mutually agreeable date and time	Send reminder of upcoming discussion via email, call, or text Send clear instructions to access the call via phone or computer
Logistics	Requires sufficient space to facilitate a group discussion of 8-10 participants and additional staff Requires participants to travel to specified locations (with consideration for travel reimbursement, public transport, and safety)	Requires telephone or Internet access among participants and staff Requires consideration of where and how participants connect online or by phone in terms of safety and privacy	Clear instructions provided during telephone screening and sent via email with screenshots Utilize notetaker to help troubleshoot any connection challenges that participants experience
Description of focus groups	Generally understood by participants; often viewed as similar to support groups	Described as *online* focus groups, but less understood and potentially daunting for participants with lower technological literacy	Clarify that individuals can participate by logging in online (with or without video) or calling in via telephone
Data security	Focuses on security of audio recording and transcripts	Focuses on security of audio recording within the electronic system and transcripts	Continue to monitor platform for any changes to data security features
Privacy	Participants requested not to disclose their real names and use pseudonyms Participants may recognize each other from the community	Participants requested not to disclose their real names and use pseudonyms; participants given the option to turn their Web cameras on or off Participants may recognize each other from the online community	Host permissions on the back end allow staff to change the name of participants who fail to use pseudonyms
Rapport	Developed during screening, consent, and throughout the discussion	May be more limited due to impersonal conference call-style setting	The use of video by study staff allows participants to view the staff and facilitates building of rapport
Participant engagement	Facilitator observes body language to guide the conversation and attempts to engage participants who do not participate in the discussion	Body language is only visible for participants who elect to utilize their cameras, though facilitator attempts to engage participants who do not participate in the discussion	Facilitator may observe mood and emotion from participants’ tone of voice to guide the conversation
Incentives	Can be provided in person in physical or electronic format	Geographic distance requires use of electronic payment or gift card or mailing the physical gift card or check	Consider local preferences for cash, mailed debit cards, or electronic payment methods
Recording and transcription	Recording may be of poor quality if participants are seated far away from each other, if participants interrupt each other, or if only one microphone is positioned in the middle of a large space; cost of transcription may increase due to poor audio quality	Software provides optional recording; cost of transcription may increase due to feedback and poor connection	Remind participants of alternative method to connect if connection quality is poor
Cost	Cost associated with space, transport, refreshments, and participant incentives	Cost associated with Zoom subscription (US $10/month) and participant incentives	Consider participant access to phone and computer

^a^CMC: computer-mediated communication.

### Comparison With Prior Work

While this study did not directly compare the quality of CMC focus group discussions to in-person focus group discussions, we did not perceive that any less information was shared in CMC focus group discussions when compared to prior experiences with in-person focus group discussions with transgender women. A qualitative study by Woodyatt and colleagues directly compared in-person focus group discussions to typed CMC focus group discussions with gay and bisexual men [16] and provides further insight into the differences between CMC and in-person focus group discussions. They found that the anonymous setting of the typed CMC discussions allowed those participants to discuss sensitive topics of intimate partner violence more candidly than those participating in person [16]. Woodyatt and colleagues, however, also reported observing a greater number of group conflicts during the typed CMC discussions compared to in-person focus group discussions; they attributed this to the complete anonymity of the typed CMC discussions [16].

Reflecting on these other variations of CMC focus group discussions, the use of synchronous CMC discussions with audio and video, as in our study with transgender women, creates a form of focus group discussion that falls on the spectrum between in-person and typed CMC focus group discussions. Providing an anonymous setting allows for open discussion of sensitive topics that are also observed with typed CMC discussions, but the audio and participant-controlled settings that allow participants to enable video may facilitate social connection and minimize group conflicts. While participants may disagree with one another’s perspectives, no group conflicts in terms of insults or heated disagreements were observed in this study.

Response rates in qualitative research among transgender women are highly variable, if reported at all [25], and were no different for this study. The participation rate in this study was moderate, with 41 of 70 (59%) candidate participants attending their scheduled discussion. Follow-up with candidates who had not attended their scheduled calls suggested that scheduling conflicts were the greatest source of their absence, with fewer absences reportedly due to technology issues. The capability to connect to the discussion through multiple mediums likely alleviated the technological barriers that may have been associated with a platform requiring participants to connect only through one medium (ie, phone or Internet). These capabilities also overcome limitations associated with typed CMC focus group discussions that would restrict participation to individuals with higher levels of literacy and typing abilities, as well as those with access to computers [17,19,26]. Nonetheless, participants with limited technological literacy or inconsistent access to a phone and/or cellular data or service, as well as circumstances necessitating immediate cash incentives, may require additional support and accommodation when connecting to CMC focus group discussions.

The preference of participants in Miami to convene in person for the CMC focus group discussion highlighted the importance and underestimated role of participant incentives. Institutional administrative requirements and service costs of other electronic payment methods led us to select the use of Amazon gift cards, which do not have additional service fees and are easy to track for reporting requirements. We frequently had to explain the Amazon business model and types of retail to participants across sites, in efforts to correct perceptions that Amazon is an online bookstore and to inform participants of the various ways in which purchases could be sent to an individual. The participants from Miami preferred to be paid directly in cash, however, for more immediate use or because they did not have reliable Internet connection to make purchases or stable housing to receive packages. The format and amount of participant incentives are as important as any other decision that is made in the implementation of focus group discussions, given their potential impact on selection bias. However, the use of CMC focus group discussions restricts the available options, as physical incentives cannot be provided directly to participants. For study populations that have greater, more immediate financial needs and less of an understanding of, or lower access to, electronic payment methods, identifying the appropriate incentive requires careful consideration and input from key community informants.

### Limitations

This study describes the feasibility of incorporating new technology into qualitative research for the purposes of improving access, reducing logistical constraints, and convening diverse participants across geographic space. Further research is needed, however, to fully articulate the benefits of this method in terms of efficiency, participant experiences, and data quality associated with this method. Technological literacy was not assessed among participants at enrollment. Incorporating a screening tool to assess technological literacy would provide important information in terms of potential biases associated with this method. To our knowledge, brief screening tools to measure technological literacy for public health research are not currently available, but are urgently needed for this research, as well as for the rapidly expanding use of technology in research methodologies.

### Conclusions

Synchronous CMC focus group discussions using a secure online conferencing system provide a new methodologic tool for qualitative research among hard-to-reach participants and those residing across geographically distant settings. These focus group discussions draw on the privacy benefits offered by typed CMC focus groups, as well as the collegiality of group dynamics offered by in-person focus group discussions. As with all focus group discussions, establishment of ground rules and employing a skilled facilitator is critical to the success of the discussion. Unique to CMC focus group discussions, we found that it is also critical to hire a notetaker who is also technologically skilled to troubleshoot and provide support to participants as they connect to the focus group discussions. Researchers who employ synchronous CMC focus group discussions with audio and video should also consider technological literacy, acceptability of noncash incentives, feasibility of distributing incentives, and imperceptibility of nonverbal communication during implementation planning. For study participants with limited time or concerns related to meeting in person, synchronous CMC focus group discussions offer a valuable option to ensure that they continue to be engaged in qualitative research.

## Abbreviations

CMC: computer-mediated communication
FIC: Fogarty International Center
HIPAA: Health Insurance Portability and Accountability Act
mPI: multiple Principal Investigator
N/A: not applicable
NCI: National Cancer Institute
NHLBI: National Heart, Lung, and Blood Institute
NIA: National Institute on Aging
NIAID: National Institute of Allergy and Infectious Disease
NICHD: National Institute of Child Health and Human Development
NIDA: National Institute on Drug Abuse
NIDDK: National Institute of Diabetes and Digestive and Kidney Diseases
NIGMS: National Institute of General Medical Sciences
NIMH: National Institute of Mental Health
OAR: Office of AIDS Research
SMS: short message service
